# Contemporary prognostic signatures and refined risk stratification of gliomas: An analysis of 4400 tumors

**DOI:** 10.1093/neuonc/noae164

**Published:** 2024-08-21

**Authors:** Hia S Ghosh, Ruchit V Patel, Eleanor Woodward, Noah F Greenwald, Varun M Bhave, Eduardo A Maury, Gregory Cello, Samantha E Hoffman, Yvonne Li, Hersh Gupta, Gilbert Youssef, Liam F Spurr, Jayne Vogelzang, Mehdi Touat, Frank Dubois, Andrew D Cherniack, Xiaopeng Guo, Sherwin Tavakol, Gino Cioffi, Neal I Lindeman, Azra H Ligon, E Antonio Chiocca, David A Reardon, Patrick Y Wen, David M Meredith, Sandro Santagata, Jill S Barnholtz-Sloan, Keith L Ligon, Rameen Beroukhim, Wenya Linda Bi

**Affiliations:** Department of Neurosurgery, Brigham and Women’s Hospital, Harvard Medical School, Boston, Massachusetts, USA; Department of Neurosurgery, Brigham and Women’s Hospital, Harvard Medical School, Boston, Massachusetts, USA; Department of Neurosurgery, Brigham and Women’s Hospital, Harvard Medical School, Boston, Massachusetts, USA; School of Medicine, Stanford University, Palo Alto, California, USA; Department of Neurosurgery, Brigham and Women’s Hospital, Harvard Medical School, Boston, Massachusetts, USA; Harvard/MIT MD-PhD Program, Harvard Medical School, Boston, Massachusetts, USA; Department of Neurosurgery, Brigham and Women’s Hospital, Harvard Medical School, Boston, Massachusetts, USA; Department of Neurosurgery, Brigham and Women’s Hospital, Harvard Medical School, Boston, Massachusetts, USA; Department of Neurosurgery, Brigham and Women’s Hospital, Harvard Medical School, Boston, Massachusetts, USA; Department of Medical Oncology, Dana-Farber Cancer Institute, Boston, Massachusetts, USA; Broad Institute of Harvard and MIT, Cambridge, Massachusetts, USA; Department of Medical Oncology, Dana-Farber Cancer Institute, Boston, Massachusetts, USA; Broad Institute of Harvard and MIT, Cambridge, Massachusetts, USA; Department of Medical Oncology, Dana-Farber Cancer Institute, Boston, Massachusetts, USA; Department of Radiation and Cellular Oncology, University of Chicago Pritzker School of Medicine, Chicago, Illinois, USA; Department of Pathology, Dana-Farber Cancer Institute, Boston, Massachusetts, USA; Sorbonne Université, Inserm, CNRS, UMR S 1127, Institut du Cerveau, ICM, AP-HP, Hôpitaux Universitaires La Pitié Salpêtrière - Charles Foix, Service de Neurologie 2-Mazarin, Paris, France; Department of Neurology, Brigham and Women’s Hospital, Boston, Massachusetts, USA; Department of Medical Oncology, Dana-Farber Cancer Institute, Boston, Massachusetts, USA; Division of Cancer Biology, Dana-Farber Cancer Institute, Boston, Massachusetts, USA; Broad Institute of Harvard and MIT, Cambridge, Massachusetts, USA; Department of Medical Oncology, Dana-Farber Cancer Institute, Boston, Massachusetts, USA; Broad Institute of Harvard and MIT, Cambridge, Massachusetts, USA; Department of Neurosurgery, Peking Union Medical College Hospital, Beijing, China; Department of Neurosurgery, University of Oklahoma Health Sciences Center, Oklahoma City, Oklahoma, USA; Department of Pathology, Dana-Farber Cancer Institute, Boston, Massachusetts, USA; Division of Cancer Epidemiology and Genetics, National Cancer Institute, Bethesda, Maryland, USA; Department of Pathology, Brigham and Women’s Hospital, Boston, Massachusetts, USA; Department of Pathology, Brigham and Women’s Hospital, Boston, Massachusetts, USA; Department of Neurosurgery, Brigham and Women’s Hospital, Harvard Medical School, Boston, Massachusetts, USA; Center for Neuro-Oncology, Dana-Farber Cancer Institute, Boston, Massachusetts; Center for Neuro-Oncology, Dana-Farber Cancer Institute, Boston, Massachusetts; Department of Pathology, Brigham and Women’s Hospital, Boston, Massachusetts, USA; Department of Pathology, Brigham and Women’s Hospital, Boston, Massachusetts, USA; Center for Biomedical Informatics and Information Technology, National Cancer Institute, Bethesda, Maryland; Division of Cancer Epidemiology and Genetics, National Cancer Institute, Bethesda, Maryland, USA; Department of Pathology, Brigham and Women’s Hospital, Boston, Massachusetts, USA; Department of Pathology, Dana-Farber Cancer Institute, Boston, Massachusetts, USA; Broad Institute of Harvard and MIT, Cambridge, Massachusetts, USA; Department of Cancer Biology, Dana-Farber Cancer Institute, Boston, Massachusetts; Center for Neuro-Oncology, Dana-Farber Cancer Institute, Boston, Massachusetts; Broad Institute of Harvard and MIT, Cambridge, Massachusetts, USA; Department of Neurosurgery, Brigham and Women’s Hospital, Harvard Medical School, Boston, Massachusetts, USA

**Keywords:** astrocytoma, glioma, molecular classification, oligodendroglioma, prognosis

## Abstract

**Background:**

With the significant shift in the classification, risk stratification, and standards of care for gliomas, we sought to understand how the overall survival of patients with these tumors is impacted by molecular features, clinical metrics, and treatment received.

**Methods:**

We assembled a cohort of patients with histopathologically diagnosed glioma from The Cancer Genome Atlas (TCGA), Project Genomics Evidence Neoplasia Information Exchange, and Dana-Farber Cancer Institute/Brigham and Women’s Hospital. This incorporated retrospective clinical, histological, and molecular data alongside a prospective assessment of patient survival.

**Results:**

Of 4400 gliomas were identified: 2195 glioblastomas, 1198 *IDH1/2*-mutant astrocytomas, 531 oligodendrogliomas, 271 other *IDH1/2*-wild-type gliomas, and 205 pediatric-type glioma. Molecular classification updated 27.2% of gliomas from their original histopathologic diagnosis. Examining the distribution of molecular alterations across glioma subtypes revealed mutually exclusive alterations within tumorigenic pathways. Non-TCGA patients had significantly improved overall survival compared to TCGA patients, with 26.7%, 55.6%, and 127.8% longer survival for glioblastoma, *IDH1/2-*mutant astrocytoma, and oligodendroglioma, respectively (all *P* < .01). Several prognostic features were characterized, including *NF1* alteration and 21q loss in glioblastoma, and *EGFR* amplification and 22q loss in *IDH1/2-*mutant astrocytoma. Leveraging the size of this cohort, nomograms were generated to assess the probability of overall survival based on patient age, the molecular features of a tumor, and the treatment received.

**Conclusions:**

By applying modern molecular criteria, we characterize the genomic diversity across glioma subtypes, identify clinically applicable prognostic features, and provide a contemporary update on patient survival to serve as a reference for ongoing investigations.

**Key Points**

- Molecular criteria shifted >25% of gliomas in their final histopathologic classification.
- Contemporary glioma cohorts show increased survival compared to TCGA.
- Molecular features identify aggressive glioma subtypes such as *EGFR-*amplified astrocytoma.

**Importance of the Study**

With the shifts in standard-of-care treatments and molecular stratification, contemporary survival data for patients with gliomas is lacking. Through one of the largest real-world multi-institutional cohorts to date, this study integrates molecular data from clinical assays, enabling us to probe salient prognostic clinical and molecular features for risk prognostication, all within the context of current therapeutics. We leverage this group of patients to demonstrate how molecular classification has significantly shifted gliomas in their final histopathologic classification, with increases in median overall survival across glioblastoma, *IDH1/2-*mutant astrocytoma, and *IDH1/2*-mutant oligodendroglioma. Further, we show how molecular markers can be integrated alongside clinical variables to refine prognostication of overall patient survival. The exponential growth of clinical trials for glioma requires such updated benchmarks for clinical outcomes alongside a framework to help interpret molecular data and treatment options.

The classification of gliomas, the most common malignant brain tumor in adults, has undergone significant transformation with routine incorporation of molecular markers, which improve prediction of tumor behavior, response to therapy, and patient outcomes.^1–6^ These molecular features were initially elucidated through large-scale efforts, including The Cancer Genome Atlas (TCGA), which were conducted before changes in treatment standards of care and the introduction of current classification schemes.^7–9^ Subsequent large-scale investigations have probed the molecular underpinnings of specific glioma subtypes or developed datasets that focus on particular molecular or clinical features.^10–15^ In our study, we aimed to curate and leverage a large, clinically heterogeneous, multi-institutional dataset of molecularly annotated gliomas to evaluate trends in patient survival and identify prognostic molecular alterations that can refine patient risk stratification. Our integrated analysis highlights significant clinical and molecular differences between contemporary patient cohorts and historical ones, distinct mutational profiles across different glioma subtypes and patient lifespan, and subtype-dependent characteristics that impact overall survival.

## Materials and Methods

### Patient Cohorts

Patients with clinically and molecularly annotated gliomas were derived from 3 datasets: (1) Dana-Farber Cancer Institute/Brigham and Women’s Hospital (DFCI/BWH); (2) Project Genomics Evidence Neoplasia Information Exchange (GENIE); and (3) TCGA. Clinical and molecular data for GENIE and TCGA were downloaded from online repositories while DFCI/BWH data was collected through chart review and institutional next-generation sequencing.^9,16,17^ A full description of data access, molecular profiling, and germline variant filtering can be found in Supplementary Material 1. Age was stratified into 4 categories: ≤19 years (pediatric); 20–39 years (young adult); 40–64 years (adult); and ≥65 years (older adults). Samples with incomplete genomic profiles or duplicates across cohorts were removed. When multiple glioma sample entries existed per patient, the earliest occurring sample was selected. This study was approved by the Institutional Review Board of the Dana-Farber/Harvard Cancer Center.

### Glioma Classification and Grade

Gliomas were classified into 5 subgroups based on molecular criteria outlined in the WHO 2021 guidelines and cIMPACT-NOW Updates 1–6: glioblastoma, astrocytoma, oligodendroglioma, pediatric-type gliomas, and other gliomas.^5,6^ Glioblastomas were *isocitrate dehydrogenases 1/2* (*IDH1/2)*-wild-type gliomas with accompanying glioblastoma-associated molecular alterations including *TERT* promoter mutation, *EGFR* copy number amplification, and/or combined whole chromosome 7 gain/chromosome 10 loss (7+/10-). Astrocytomas were *IDH1/2*-mutant gliomas without codeletion of chromosomal arms 1p and 19q. Oligodendrogliomas were *IDH1/2*-mutant gliomas with chromosome 1p19q codeletion. If 1p19q status was not available in an *IDH1/2-*mutant glioma, the presence of *ATRX* or *TP53* mutations indicated it was likely an astrocytoma.^18^ 98.8% of mutations in *IDH1/2* were either *IDH1*^*R132*^ or *IDH2*^*R172*^, while the remainder were non-canonical mutations. Low-grade pediatric-type gliomas were *IDH1/2*-wild-type gliomas with *MAPK* alterations and no glioblastoma-specific alterations. High-grade pediatric-type gliomas were *IDH1/2*-wild-type with *H3K27* or *H3G34* mutation. Finally, other *IDH1/2*-wild-type gliomas included the remaining *IDH1/2*-wild-type and diffuse astrocytic gliomas as well as those labeled “not elsewhere classified (NEC).”^19^

Following molecular reclassification, glioma grading was determined by the WHO 2021 and cIMPACT-NOW Updates 1–6. Per the criteria, all glioblastomas were designated as grade 4. *IDH1/*2-mutant oligodendrogliomas were classified as grade 3 and *IDH1/2*-mutant astrocytomas as grade 4 if they had a homozygous deletion of *cyclin-dependent kinase inhibitor 2A* and *2B* (*CDKN2A/B*).^5^ As there are no listed molecular criteria to distinguish grade 2 from grade 3 *IDH1/2-*mutant astrocytoma, these grades were assigned based on the clinically annotated grade. Grade 1 gliomas, when present, were also designated using their original annotated grade for pediatric-type and other *IDH1/2*-wild-type gliomas.

### Molecular Variant Prevalence

Variants of interest were selected if assayed in at least 1 of the 3 glioma cohorts. Variant prevalence was calculated based on the total number of samples assayed for that gene. To capture a broad set of variants, we selected mutations, copy number variants (CNV), structural variants, or arm-level changes with >1% prevalence. Statistical comparison of molecular alteration prevalence across age groups was performed using Chi-square and pair-wise proportion tests with Holm-Bonferroni correction at a significance level of *P* < .05.

### Molecular Correlations and Genomic Distance

Correlations between molecular alterations and clinical variables were examined within glioblastoma, *IDH1/2-*mutant astrocytoma, and oligodendroglioma using the Fisher’s Exact test. *IDH1/2* was excluded as a feature as it was used to define glioma subtypes. Given the multiple genomic alterations explored, we used permutation testing with a Curveball algorithm to generate null molecular alteration matrices which enabled statistical comparison between individual molecular features.^20,21^ CNV and mutations were treated independently to generate the null alteration matrix. Clinical and molecular correlations were significant after corrections for multiple comparisons using the false discovery rate approach with significance *q* < 0.1. Genomic distance within and between glioma subtypes for gene mutations was quantified using the Jaccard similarity index: a Jaccard Distance (JD) of 1 indicates the greatest genomic difference.^22^ Statistical comparison of JDs was performed using analysis of variance (ANOVA) with post hoc Tukey’s test at a significance level of *P* < .05.

### Survival Analysis

Overall survival and prognostic features were examined in primary glioblastoma, *IDH1/*2-mutant astrocytoma, and oligodendroglioma for patients ≥20 years old. All patients in the TCGA cohort were diagnosed or operated on between 1989 and 2013 while the majority (97.6%) of patients in the non-TCGA cohort (DFCI/BWH and GENIE) were diagnosed or operated on between 2006-2020 (Supplementary Material 2). The date of diagnosis was only used if the date of surgery was not available. Kaplan–Meier curves were generated to compare survival between glioma groups, with significance deemed when *P* < .05. The upper limits of survival ranges were the time at which all patients were deceased or when the last follow-up was completed.

To determine significant prognostic features for overall survival, an initial univariate Cox analysis was separately performed for glioblastoma, *IDH1/*2-mutant astrocytoma, and oligodendroglioma in the non-TCGA cohort. As the extent of tumor resection data was not available from the GENIE and TCGA datasets, this was not included as a covariate across all gliomas. Given the documented significance of *MGMT*-methylation on treatment response, glioblastoma samples were selected only if *MGMT*-methylation status was known. For univariate analysis, molecular features were selected as significant if *q* < 0.2 after multiple comparisons correction and prevalence was >3.5% within the glioma type assessed. Features selected as significant on univariate analysis were passed to a multivariate Cox regression model performed for each glioma subtype, along with additional clinical features (patient age, sex, race, receipt of chemotherapy, and tumor grade (if applicable)). A stepwise backward elimination approach was performed to prevent overfitting and remove any features with undefined confidence intervals. Adjusted features were significant if *P* < .05. Internal validation was performed using subsets of the non-TCGA dataset across 15 folds. Each fold randomly sampled 65% of the dataset without replacement. Pseudo-R^2^ values (RD) were computed for each multivariate model and compared to the model generated on the entire dataset. Significant prognostic features were integrated into nomograms predicting patient survival for glioma subtypes. Nomograms were constructed on DFCI data as the extent of resection and radiation therapy status were not available in the GENIE dataset.

## Results

### Molecular Features Refine Histopathological Diagnoses

**Figure 1.**
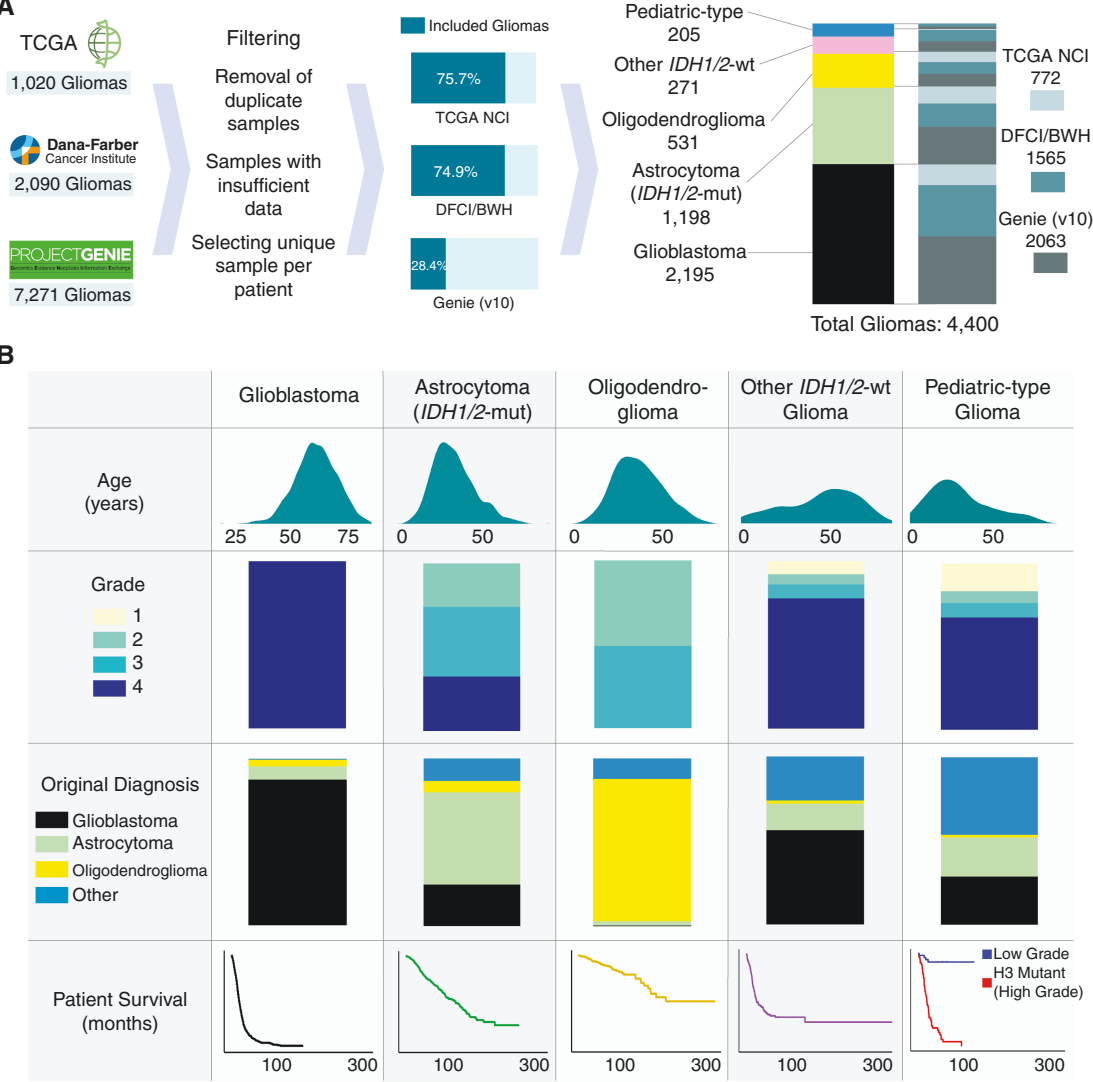
Cohort overview. (A) Overview of constructing a pooled molecularly annotated glioma cohort. (B) Summary of the pooled glioma cohort.

We identified 4400 unique patients (median age 52 years, range 0–94 years) with molecularly annotated gliomas from 3 datasets: DFCI/BWH (*n* = 1565), GENIE (*n* = 2063), and TCGA (*n* = 772; Figure 1A; Table 1). This spanned 2195 glioblastoma, 1198 *IDH1/2*-mutant astrocytoma, 531 *IDH1/2*-mutant oligodendroglioma, 271 other *IDH1/2-*wild-type glioma, and 205 pediatric-type glioma (89 low-grade, 116 high-grade), all classified according to the World Health Organization (WHO) Classification of Tumors of the Central Nervous System 2021 guidelines and the 6 cIMPACT-NOW Updates.^5,6^

**Table 1.** Cohort

Variable	Glioblastoma	Astrocytoma (*IDH1/2-*mut)	Oligodendro-glioma	Pediatric-type Glioma	Other *IDH1/2*-wild-type gliomas
Patients, *n*	2195	1198	531	205	271
*Cohort, n (%)*					
TCGA NCI	325 (14.8)	266 (22.2)	164 (30.9)	5 (2.4)	12 (4.4)
DFCI/BWH	791 (36.0)	358 (29.9)	176 (33.1)	96 (46.8)	144 (53.1)
Genie (v10)	1079 (49.2)	574 (47.9)	191 (36.0)	104 (50.7)	115 (42.4)
Sex (female), *n* (%)	886 (40.4)	483 (40.3)	250 (47.2)	95 (46.3)	115 (42.4)
Median age, years (range)	61 (6-94)	36 (7-90)	43 (13-81)	27 (1-78)	51 (0-90)
*Age (years), n (%)*					
≤19	5 (0.2)	26 (2.3)	6 (1.1)	50 (31.1)	32 (12.5)
20–39	58 (2.7)	673 (56.6)	193 (36.5)	69 (42.9)	51 (20.0)
40–64	1301 (59.6)	455 (38.2)	290 (54.8)	30 (18.6)	111 (43.5)
≥65	818 (37.5)	35 (2.9)	40 (7.6)	12 (7.5)	61 (23.9)
Race (White), *n* (%)	1918 (93.3)	1009 (91.9)	463 (92.6)	139 (82.2)	208 (86.7)
*Histopathologic diagnosis, n (%)*					
Glioblastoma	1918 (87.4)	297 (24.8)	2 (0.4)	59 (28.8)	152 (56.1)
Astrocytoma	176 (8.0)	659 (55.0)	14 (2.6)	48 (23.4)	43 (15.9)
Oligodendroglioma	13 (0.6)	81 (6.8)	450 (84.8)	3 (1.5)	5 (1.8)
Other gliomas	88 (4.0)	161 (13.4)	65 (12.2)	95 (46.3)	71 (26.2)
*Grade, n (%)*					
1	—	—	—	21 (16.7)	16 (8.0)
2	—	266 (25.9)	240 (51.1)	9 (7.1)	12 (6.0)
3	—	427 (41.5)	230 (48.9)	11 (8.7)	17 (8.5)
4	2195 (100.0)	334 (32.5)	—	85 (67.5)	156 (77.6)
*Molecular alterations, n (%)*					
*TERT* promoter	1522 (91.0)	40 (6.0)	317 (94.3)	4 (2.3)	0 (0)
*EGFR* amplification	1038 (47.5)	17 (1.7)	0 (0)	6 (3.1)	0 (0)
Whole Chr7 Gain/Chr10 loss	1231 (57.7)	8 (0.8)	1 (0.2)	2 (1.1)	0 (0)
*CDKN2A/B* hom. del.	1234 (56.5)	99 (10.1)	8 (1.5)	17 (8.7)	81 (29.9)
*PDGFRA*	248 (11.3)	77 (6.4)	20 (3.8)	33 (16.3)	40 (14.8)
*PTEN*	1046 (47.7)	40 (3.3)	12 (2.3)	12 (5.9)	67 (24.7)
*ATRX*	58 (2.6)	733 (61.3)	34 (6.4)	65 (32.5)	55 (20.3)
*TP53*	562 (25.6)	1099 (91.7)	40 (7.5)	77 (37.6)	108 (39.9)

Summary table of surveyed gliomas after molecular classification. Calculated percentages for clinical variables or molecular alterations are out of the total number of samples included or assayed per variable. Chr, chromosome; n, number.

Molecular classification significantly refined glioma subtypes from their original histopathological diagnoses. Among the molecularly classified glioblastomas, 87.4% were consistent with their original designation (Figure 1B, Table 1). Molecularly defined *IDH1/2*-mutant astrocytomas (grades 2–4) showed the greatest heterogeneity in their original histopathologic classifications—55.0% were previously classified as astrocytomas, 24.8% as glioblastoma, 13.4% as other gliomas, and 6.8% as oligodendrogliomas. By contrast, *IDH1/2*-mutant 1p/19q codeleted oligodendrogliomas showed higher concordance with their histopathologic designation (84.8% originally classified as oligodendrogliomas). Pediatric-type gliomas were enriched in glioblastoma (28.8%) and astrocytoma (23.4%) while other *IDH1/2*-wild-type gliomas were largely histologically characterized as glioblastoma (56.1%).

### Molecular Alterations Vary Across Glioma Subtypes

**Figure 2.**
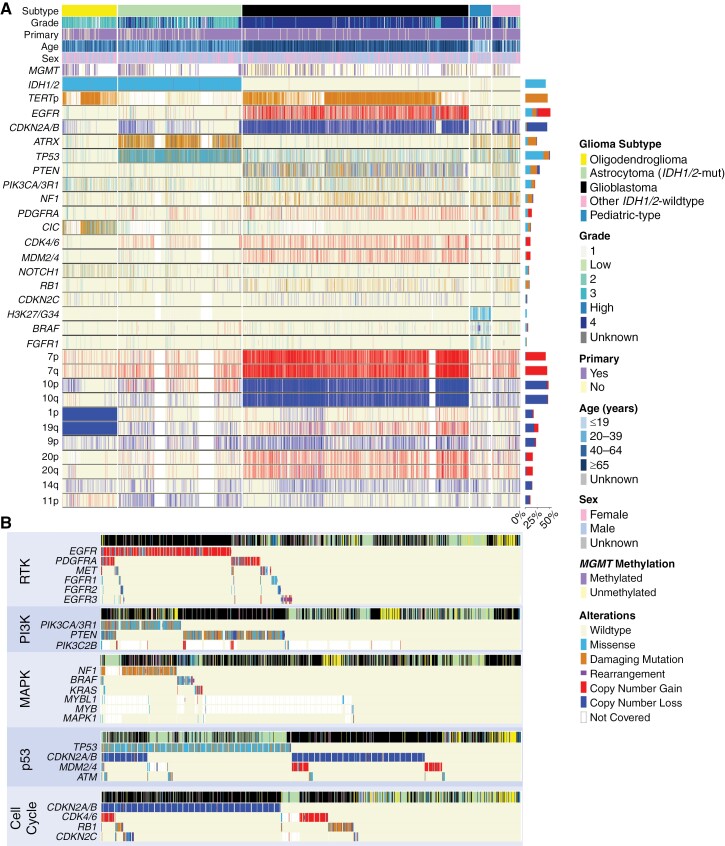
Mutational signatures in glioma. (A) Mutational signatures of gliomas stratified by tumor subtype. Genes were filtered out of the comutation plot if alteration prevalence was low or if not involved in molecular glioma classification guidelines. (B) Molecular alterations within the same tumorigenic pathway were frequently mutually exclusive in glioma.

Trends emerged alongside canonical molecular alterations in the dominant glioma subtypes (Figure 2A, Table 1, Supplementary Material 3). For example, while a majority of glioblastoma had whole chromosome 7 gain/chromosome 10 loss (7+/10−) (57.7%), partial 7+/10− alterations were found in an additional 31.1% of glioblastomas. When whole 7+/10− were observed in non-glioblastoma subtypes, it was exclusively present in grade 3 and 4 tumors (grade 3: 4 tumors, grade 4: 7 tumors). In *IDH1/2*-mutant astrocytomas, *EGFR* amplifications were rare (1.7%) but confined to higher-grade tumors (grade 3: 5 tumors, grade 4: 12 tumors). Though *IDH1/*2-mutant oligodendrogliomas had frequent alterations in *TERT* promoter (94.3%) and *CIC* (69.1%), there remained a low prevalence of *TP53* (7.5%) and *ATRX* (6.4%) alterations. Low-grade pediatric-type gliomas were enriched with *BRAF* mutations and rearrangements (39.3% and 25.8%, respectively) and *FGFR1* alterations (30.3%), while *TP53* alterations (39.9%) and homozygous deletion of *CDKN2A/B* (29.9%) were prevalent in other *IDH1/2*-wild-type gliomas.

We next assessed 5 frequently altered pathways associated with tumorigenesis: receptor tyrosine kinase (RTK), phosphoinositide-3-kinase (PI3K), mitogen-activated protein kinase (MAPK), p53, and cell cycle. Gliomas frequently exhibited concurrent aberrations in these pathways, with glioblastoma having an average of 2.8 pathways affected (median: 3), *IDH1/2*-mutant astrocytoma with 1.7 (median: 1), and oligodendroglioma with 1.5 (median: 1, Supplementary Material 4). However, gliomas rarely harbored multiple alterations within each pathway, a phenomenon commonly observed in cancer genomes (Figure 2B).^23^ Amongst RTKs, alterations in *EGFR* showed limited cooccurrence with *PDGFRA* (8.4%), *MET* (4.6%), and *FGFR1-3* (2.7%, 2.4%, 2.4%, all *P* < .01). Of all RTKs analyzed, *EGFR* had the highest prevalence of rearrangement. In the PI3K pathway, there was minimal cooccurrence of *PIK3CA/3R1* and *PTEN* mutations (8.3%, *P* < .01), especially in glioblastoma. Across the MAPK pathway, mutations in *NF1*, *BRAF*, and *KRAS* were almost entirely mutually exclusive: mutations in *NF1* cooccurred with *BRAF* in 3.6% and with *KRAS* in 2.2% of altered cases, while *BRAF* and *KRAS* mutations cooccurred in 1.3% of altered cases (all *P* < .01). In the p53 pathway, while *TP53* and *CDKN2A/B* alterations overlapped in a subset of glioblastomas and *IDH1/2*-mutant astrocytomas, *TP53* mutation predominated in *IDH1/2*-mutant astrocytomas, while *CDKN2A/B* alterations predominated in glioblastomas. Additionally, focal amplifications of *MDM2 or MDM4*, known regulators of p53, were also seen in a subpopulation of glioblastomas without *TP53* or *CDKN2A/B* alteration. Finally, amongst other cell cycle mediators, there was minimal overlap between alterations in *CDKN2A/B*, *CDK4/6*, *RB1*, and *CDKN2C*. Interestingly, there was heterogeneity in the association of alterations between different pathways, namely when considering the cell cycle and p53 pathways. In glioblastoma, 82.8% of cases had cooccurrence of alterations between these 2 pathways, with alteration in *CDKN2A/B* impacting both the cell cycle and p53 pathways; 2.4% of glioblastoma had an alteration only in the cell cycle pathway and 4.8% only in the p53 pathway. This diverged from *IDH1/2*-mutant astrocytoma (28.8% of cases demonstrating cooccurrence, 0.5% cell cycle pathway only, 55.1% p53 pathway only) and *IDH1/2*-mutant oligodendroglioma (7.9% cooccurrence, 4.3% cell cycle pathway only, 7.5% p53 pathway only).

### Genomic Correlates and Distance Distinguish Glioma Subtypes

**Figure 3.**
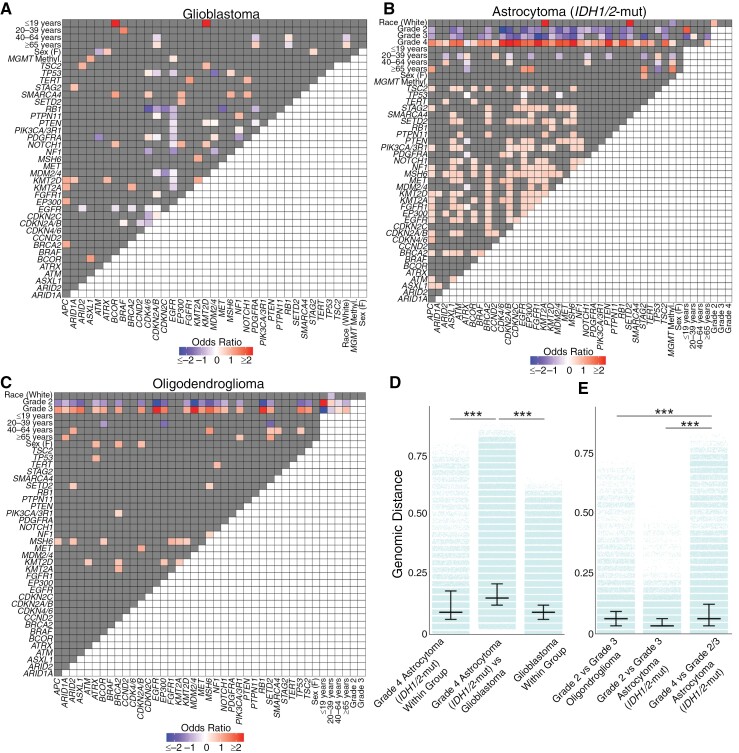
Genomic correlations in glioma subtypes. Heatmap of positively and inversely correlated genes for (A) glioblastoma, (B) *IDH1/*2-mutant astrocytoma, and (C) oligodendroglioma. (D) The genomic distance between grade 4 *IDH1/2-*mutant astrocytomas and glioblastoma was greater than the genomic distance within each glioma group. (E) Grade 4 *IDH1/2*-mutant astrocytoma were more genomically distinct from Grade 2/3 *IDH1/2*-mutant astrocytoma, with greater genomic separation compared to grade 2 and grade 3 *IDH1/2*-mutant astrocytoma or between grade 2 and grade 3 oligodendroglioma. *P* < .001 (***).

When we stratified the glioma subtypes, we observed distinct relationships between clinical variables and molecular alterations (Figure 3A-C). In glioblastoma, alterations in *CDKN2A/B* and *PDGFRA* were significantly enriched in patients ≥65 years old while there was a depletion of these alterations in patients between 40 and 64 years old (Figure 3A). *RB1* alterations showed the inverse relationship, with enrichment in patients between 40 and 64 years old. Age also showed associations with molecular alterations in *IDH1/2*-mutant astrocytomas and oligodendrogliomas, where patients <40 years old exhibited distinct correlated molecular alterations compared to those between 40 and 64 and ≥65 years (Figure 3B-C). Moreover, there was a clear genomic distinction across different grades within *IDH1/2-*mutant astrocytomas and oligodendrogliomas. Grade 4 *IDH1/2*-mutant astrocytomas and grade 3 oligodendrogliomas showed positive correlations with a range of molecular alterations, while grades 2/3 in *IDH1/2*-mutant astrocytomas and grade 2 oligodendrogliomas were negatively correlated with nearly all of the same molecular alterations. Relationships between molecular alterations also emerged: notably, *EGFR* alterations were positively correlated with both *CDKN2A/B* and *PDGFRA* alterations in *IDH1/2*-mutant astrocytoma, reflecting the cooccurrence of canonical glioblastoma molecular alterations in these tumors.

We quantified the heterogeneity of glioma genomes using genomic distance. As expected, we observed a greater genomic variability between gliomas of different subtypes than within a subtype (Supplementary Material 5). Specifically, the genomic difference between glioblastoma and *IDH1/2*-mutant astrocytoma (median JD: 0.147) was significantly higher than the genomic heterogeneity within glioblastoma (median JD: 0.088, *P* < .001) or *IDH1/2*-mutant astrocytoma (median JD: 0.059, *P* < .001). Similarly, the genomic distances between glioblastoma and oligodendroglioma (median JD: 0.118) and between *IDH1/2-*mutant astrocytoma and oligodendroglioma (median JD: 0.088) were greater than the genomic heterogeneity within each respective glioma subtype (all *P* < .001).

When we subdivided gliomas by grade, we saw distinct patterns of genomic distance. Grade 4 *IDH1/2*-mutant astrocytomas and glioblastomas were more genomically distant from each other (median JD: 0.147) compared to genomic differences within grade 4 *IDH1/2*-mutant astrocytomas (median JD: 0.088) or within glioblastomas (median JD: 0.088, *P* < .001, Figure 3D). Furthermore, grade 4 *IDH1/2*-mutant astrocytomas showed a greater genomic distance from grade 2/3 *IDH1/2-*mutant astrocytomas than between grades 2 and 3 *IDH1/2*-mutant astrocytomas (*P* < .001, Figure 3D). This distance between grade 4 versus grade 2–3 *IDH1/2*-mutant astrocytoma outstripped the genomic distance between grade 3 versus grade 2 oligodendrogliomas (*P* < .001, Figure 3D). This demonstrated the unique genomic makeup of grade 4 *IDH1/2*-mutant astrocytomas, distinguishing them from glioblastoma and other grade 2 and 3 *IDH1/2*-mutant gliomas.

### Glioma Cohort and Subtype Influence Survival

**Figure 4.**
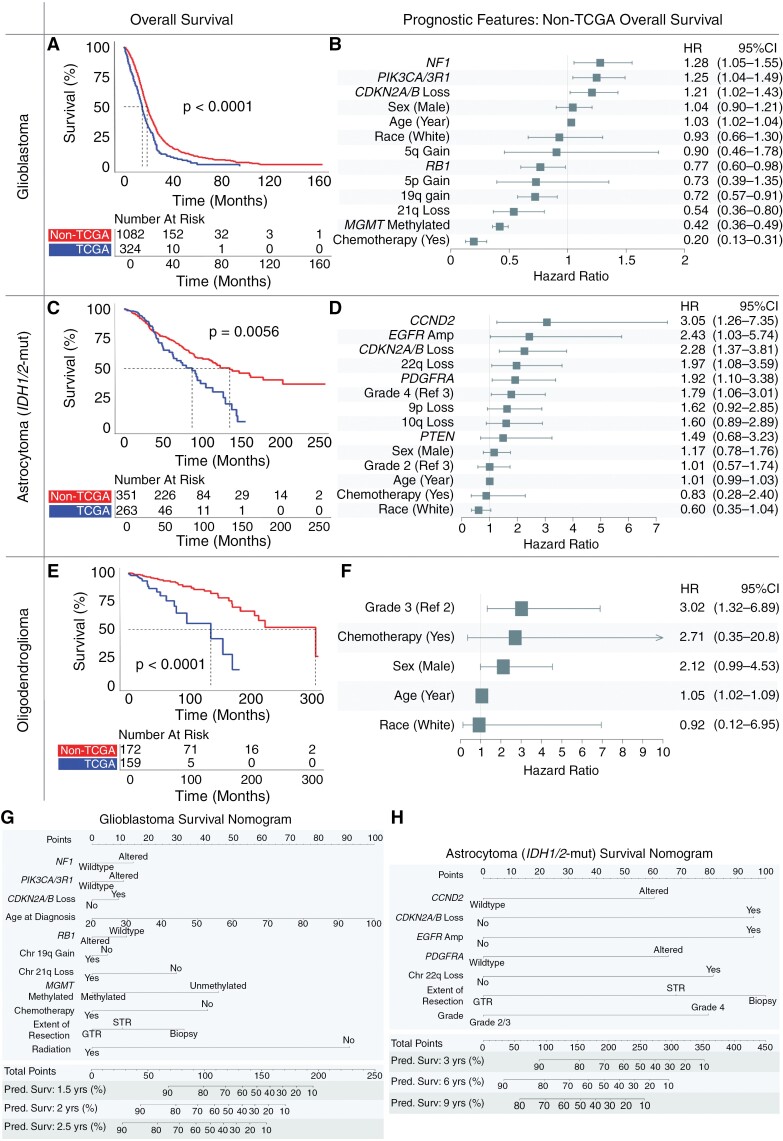
Overall survival and prognostic features in glioma subtypes. Kaplan–Meier curves demonstrate overall survival in the non-The Cancer Genome Atlas (TCGA) cohort exceeds that of the TCGA cohort for patients with newly diagnosed (A) glioblastoma, (C) *IDH1/2*-mutant astrocytoma, and (E) oligodendroglioma. Multivariate adjusted clinical and molecular features predictive of overall patient survival for newly diagnosed (B) glioblastoma, (D) *IDH1/2-*mutant astrocytoma, and (F) oligodendroglioma. Nomograms quantify the predicted probability of patient survival integrating clinical and molecular features for (G) glioblastoma and (H) *IDH1/2*-mutant astrocytoma.

We observed a significant increase in median survival between patients in the non-TCGA cohort compared to those in the TCGA cohort, despite similarities in demographic profile (Supplementary Material 6). Amongst all patients ≥20 years old with primary gliomas, the median survival varied across glioblastoma (18.0 months, range: 0.1–164.0 months), *IDH1/2-*mutant astrocytoma (118.5 months, range: 0.1–262.2 months), and oligodendroglioma (213.9 months, range: 0.1–324.4 months). For glioblastoma, non-TCGA patients had a median overall survival of 19.0 months (range: 0.2–164.0 months), which was 26.7% longer than the median overall survival of TCGA patients (15.0 months, range: 0.1–95.3 months, *P* < .001, Figure 4A). This difference in median survival between non-TCGA and TCGA cohorts was more pronounced for *IDH1/2-*mutant astrocytoma, where non-TCGA patients had a median survival of 136.8 months (range: 1.0–262.2 months), compared to 87.9 months (range: 0.1–157.1 months, *P* = .0002) in TCGA patients (Figure 4C). Median survival of grade 2/3 *IDH1/2-*mutant astrocytomas more than doubled that of grade 4 *IDH1/2-*mutant astrocytomas while grade 2 and grade 3 *IDH1/2*-mutant astrocytomas did not differ significantly (Supplementary Material 7). Similarly, in the case of oligodendroglioma, non-TCGA patients had a median survival of 307.5 months (range: 0.3–324.4 months), more than double the median survival of TCGA patients at 135.0 months (range: 0.1–183.3 months, *P* < .0001, Figure 4E), and with grade 2 oligodendroglioma patients not reaching median survival at latest follow-up (Supplementary Material 7).

### Prognostic Features Vary Across Glioma Subtypes

Clinical, molecular, and treatment characteristics each impacted prognosis across glioma subtypes on multivariate modeling within the non-TCGA cohort (Figure 4). Almost all non-TCGA patients received chemotherapy (94.8%) with 91.1% of those receiving chemotherapy having temozolomide as part of their treatment plan.

In non-TCGA patients with glioblastoma, receipt of chemotherapy (HR: 0.20), methylated *MGMT* (HR: 0.42), 21q loss (HR: 0.54), 19q gain (HR: 0.72), and *RB1* alteration (HR: 0.77) positively affected survival. *NF1* alteration (HR: 1.28), *PIK3CA/3R1* alteration (HR: 1.25), *CDKN2A/B* loss (homozygous or heterozygous, HR: 1.21), and increasing age (HR: 1.03) negatively impacted survival for glioblastoma (Figure 4B). Homozygous and heterozygous loss of *CKDN2A/B* were assessed together as both exerted a similar negative effect on overall survival (Supplementary Material 8A). Chromosome 21q loss emerged as a novel positive prognostic feature, with overall survival diverging around the 2-year mark (Supplementary Material 8B). To validate these features in the context of additional treatment data, multivariate testing was performed in the DFCI cohort, where the extent of surgical resection (EOR) and radiation (RT) data was available. Even after adjusting for EOR and RT in this institutional cohort, almost all molecular features remained significant, including *NF1, PIK3CA/3R1, RB1,* and 21q loss (Supplementary Material 9A). Gross-total resection positively affected survival (vs sub-total resection, HR: 0.78) while biopsy only (vs sub-total resection, HR: 1.66) negatively affected survival.

In non-TCGA *IDH1/2-*mutant astrocytomas, *CCND2* alteration (HR: 3.05), *EGFR* amplification (HR: 2.43), *CDKN2A/B* loss (homozygous or heterozygous loss, HR: 2.28), 22q loss (HR: 1.97), and *PDGFRA* alteration (HR: 1.92) were negatively prognostic (Figure 4D). 10q loss (HR: 1.60, *P* = .08) and 9p loss (HR: 1.62, *P* = .09) approached significance as negative prognostic features. Within the DFCI cohort, after EOR and RT adjustment, almost all of these features remained significant (Supplementary Material 9B). Gross-total resection (vs sub-total resection) was a positive prognostic feature (HR: 0.43) while biopsy (vs sub-total resection) was more variable. Consistent with prior results, tumor grade 2 versus grade 3 and *MGMT*-methylation were not significant independent prognostic features.^24,25^

Looking specifically at *EGFR* amplification, *CDKN2A/B* loss, 10q loss, and 22q loss, patients possessing at least one of these 4 features had significantly reduced survival compared to those without alteration (Supplementary Material 10). Patients with *EGFR* amplification had a median survival of 37.6 months (range: 2.3–97.1 months), which was less than one-third of the median survival for patients without *EGFR* amplification (120.6 months, range: 0.1–262.2 months, *P* < .0001, Supplementary Material 10A). Similarly, patients with homozygous loss of *CDKN2A/B* loss had a median survival of 30.0 months (range: 2.3–167.6 months) compared to 71.6 months if their tumor harbored a heterozygous loss of *CDKN2A/B* (range: 5.8–164.8 months) or 136.8 months with no loss of *CDKN2A/B* (range: 0.1-262.2 months, *P* < .0001, Supplementary Material 10B). Patients with 10q loss had a median survival of 43.9 months (range: 0.1–208.8 months), which was less than half the median survival of patients with 10q retained (124.5 months, range: 0.1–262.2 months, *P* < .0001, Supplementary Material 10C). Moreover, patients with 22q loss had a median survival of 45.3 months (range: 0.1–128.3 months), compared to 124.7 months for patients with 22q retained (range: 0.1-262.2 months, *P* < .001, Supplementary Material 10D). Notably, these negative prognostic molecular features had limited cooccurrence, with only 25.9% of patients with *EGFR* amplification, *CDKN2A/B* loss, 10q loss, and 22q loss having more than one of these features (Supplementary Material 10E).

Prognostic features in non-TCGA *IDH1/2-*mutant 1p/19q-codeleted oligodendrogliomas were rare and no molecular alterations significantly influenced survival, including *MGMT*-methylation status. High tumor grade was the strongest negative prognostic indicator (HR: 3.02), followed by increasing age (HR: 1.05, Figure 4F). This remained consistent in the DFCI cohort after adjustment for EOR and RT (Supplementary Material 9C). Internal validation of multivariate models for glioblastoma, *IDH1/2*-mutant astrocytoma, and *IDH1/2-*mutant oligodendroglioma demonstrated relative consistency in model fit, with the greatest heterogeneity observed amongst *IDH1/2*-mutant oligodendroglioma models (Supplementary Material 11).

Given the differences in overall survival between the non-TCGA and TCGA cohorts, we examined features identified above across the whole dataset to determine what may be driving cohort differences, including cohort status (non-TCGA or TCGA) as a covariate. In glioblastoma, molecular and clinical features remained largely stable, including *PIK3CA/3R1* alteration (HR: 1.31, *P* < .01), *CKDN2A/B* loss (HR: 1.22, *P* = .01), increasing age (HR: 1.03, *P* < .001), *RB1* alteration (HR: 0.79, *P* < .05), 19q gain (HR: 0.70, *P* < .001), 21q loss (HR: 0.55, *P* < .002), methylated *MGMT* (HR: 0.46, *P* < .001), and receipt of chemotherapy (HR: 0.37, *P* < .001). Even after controlling for these features, cohort status was significantly associated with patient survival, with non-TCGA cohort status positively prognostic for survival (HR: 0.56, *P* < .001). A similar effect was also seen for *IDH1/*2-mutant astrocytoma (non-TCGA cohort status HR: 0.55, *P* < .01) and *IDH1/2*-mutant oligodendroglioma (non-TCGA cohort status HR: 0.17, *P* < .001) even after controlling for features identified above within each glioma subtype. Clinical and molecular features for glioblastoma and *IDH1/*2-mutant astrocytoma were integrated into nomograms using data which included EOR to quantify the predicted probability of patient survival (Figure 4G-H).

### Distribution of Molecular Alterations Changes with Age

**Figure 5.**
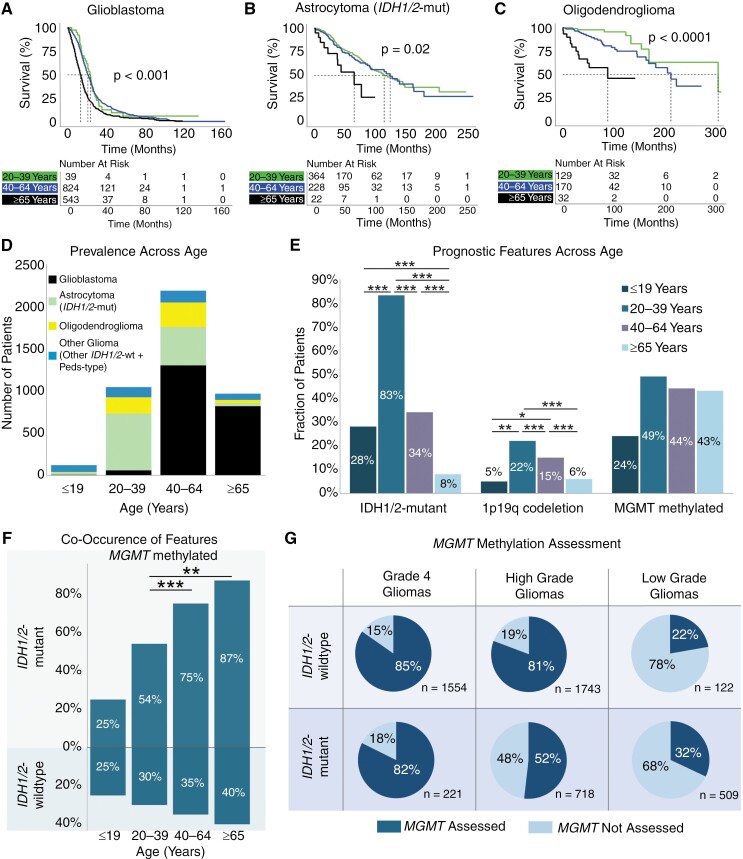
Impact of age on survival and prognostic features. Kaplan–Meier curves for glioma overall survival stratified by age for (A) glioblastoma, (B) *IDH1/2-*mutant astrocytoma, and (C) oligodendroglioma. (D) Prevalence of glioma subtypes across age categories. (E) Prevalence of *IDH1/2*-mutation, *MGMT*-methylation, and chromosome 1p/19q codeletion across age categories. (F) Cooccurrence of *MGMT*-methylation with *IDH1/2* mutation status. (G) Percent of gliomas assayed for *MGMT*-methylation stratified by *IDH1/2* mutation status and tumor grade (high: grades 3–4, low: grades 1–2). *P* < .05 (*), *P* < .01 (**), *P* < .001 (***).

Given the prognostic significance of age for glioblastoma and oligodendroglioma, we investigated overall survival in different glioma subtypes across age strata. Patients ≥65 years old with glioblastoma, *IDH1/2*-mutant astrocytoma, or oligodendroglioma had lower median survival compared to patients between 40–64 years and 20–39 years (all *P* ≤ .02, Figure 5A-C).

We subsequently surveyed 3 molecular signatures with positive prognostic significance across different age groups in glioma: *IDH1/2-*mutation, *MGMT*-methylation, and 1p19q codeletion. As expected, across all gliomas, the prevalence of *IDH1/2*-mutant gliomas decreased as patients became older, with only 75 out of 966 patients ≥65 years old having an *IDH1/2-*mutant astrocytoma or oligodendroglioma (Figure 5D-E). In contrast, the prevalence of *MGMT* promoter methylation was similar across all adult patients, ranging between 43% and 49% across all gliomas (X^2^*P* = .091, Figure 5E). Amongst all *IDH1/*2-mutant gliomas, 64.1% were *MGMT* methylated. However, among the few patients over 65 years old with *IDH1/2-*mutation, there was significantly greater cooccurrence of *MGMT*-methylation (87%) compared to patients between 20 and 39 years old (54%, Figure 5F, X^2^*P* < .001). Notably, a significant proportion of gliomas were not assessed for *MGMT*-methylation status, especially amongst *IDH1/2*-mutant tumors (Figure 5G). This was particularly evident among low-grade gliomas, where only 32% of low-grade *IDH1/2*-mutant gliomas were assessed for *MGMT*-methylation status compared to 52% of high-grade and 82% of grade 4 *IDH1/2*-mutant gliomas.

## Discussion

The integration of molecular criteria with histopathological features for glioma classification has advanced neuro-oncology care, linking changes in genotype to tumor phenotype and clinical behavior. We provide an updated overview of survival estimates across glioma subtypes, dissect pathways involved in glioma tumorigenesis, and present clinically applicable tools that integrate clinical and molecular features for prognostication. Our findings in the non-TCGA cohorts further emphasize the importance of using clinically representative datasets when developing and validating molecular biomarkers.

Stratification by more contemporary versus the TCGA cohort revealed noteworthy increases in survival rates across various glioma subtypes compared to previous estimates. For instance, patients with glioblastoma in the non-TCGA cohort had a median survival of 19.0 months, exceeding the median survival of 15.0 months observed in previous clinical trials and in the TCGA cohort.^26^ In comparison, *IDH1/2-*mutant astrocytomas and oligodendrogliomas have seen more significant increases in median survival during the contemporary period: non-TCGA *IDH1/2-*mutant astrocytoma patients had a median survival of 11.4 years while the median survival for oligodendrogliomas was 25.6 years. These survival outcomes are more than double what was seen in the TCGA cohort as well as other population-wide estimates.^27,28^ Notably, even after controlling for molecular and clinical features, cohort status remained significantly prognostic for patient survival. The drivers of these improvements in survival across glioma subtypes are likely multifactorial including increased accuracy in molecular diagnostics, recognition of surgical advantage with early intervention, evolution in technologies for safer maximal tumor resection, more widespread use of chemoradiation, and availability of investigational agents.^29–31^ Across glioblastoma, *IDH1/*2-mutant astrocytoma, and oligodendroglioma, heterogeneity in patient populations may have contributed to the differences between non-TCGA and TCGA survival estimates. As many GENIE institutions do not report survival data, survival analyses for the non-TCGA cohort only included patients from DFCI and MSKCC, 2 large, urban, research-intensive tertiary care medical centers. Nevertheless, the notable divergence in survival between patients in the non-TCGA versus TCGA cohorts indicates the importance of using contemporary patient profiles when assessing standards of care and novel therapeutics for gliomas.

Through multivariate analysis of a highly powered cohort, we identified molecular prognostic features that were specific to glioma subtypes, most of which retained significance for prognostic impact even after adjustment of extent-of-resection and RT therapy. The size of this cohort enabled us to elucidate rarer but significant molecular signatures, including 21q loss in glioblastoma as well as *EGFR* amplification and chromosome 22q loss in *IDH1/2*-mutant astrocytoma. The molecular heterogeneity underlying *IDH1/*2-mutant astrocytoma manifested in feature assessment, with several features such as *MYC* amplification and *MET* alterations significant on univariate analysis but not after multiple comparisons correction, similar to that reported as part of cIMPACT-NOW.^18,24^ We also demonstrate that heterozygous loss of *CKDN2A/B* conferred a negative impact on overall survival similar to homozygous loss of *CDKN2A/B*, suggesting prognostic value of *CDKN2A/B* in glioblastoma even with incomplete loss. Evaluating the implications of *CDKN2A/B* heterozygous loss determined through clinically used assays will be critical to ensure it is a robust marker of poor prognosis. In comparison to glioblastoma and *IDH1/2*-mutant astrocytoma, oligodendroglioma displayed no specific molecular alterations associated with prognosis.^5^ This observation suggests that higher-grade oligodendrogliomas may be characterized by a more diverse array of genomic alterations correlating with tumor grade, rather than a few dominant recurrent driver mutations dictating higher-grade behavior. Placing molecular features alongside traditional markers shown associated with survival such as increased patient age refines survival predictions and identifies patients who may benefit from early targeted therapeutics.^32,33^

Increased patient age is associated with unfavorable prognostic features, as previously demonstrated in smaller cohorts.^32,33^ Older patients were more likely to have higher-grade tumors (oligodendroglioma, and astrocytomas) and disadvantageous *PDGFRA* alterations (glioblastoma). Two more favorable features, *IDH1/2*-mutation, and 1p19q codeletion decreased as patients aged. Interestingly, the prevalence of *MGMT*-methylation was comparable across age groups. Furthermore, in the limited number of older patients who had *IDH1/2*-mutation, these individuals were significantly more likely to have cooccurring *MGMT*-methylation. This may suggest that there is a subset of patients ≥65 years old who may have a biologically more favorable subtype of glioma. However, only 32% of patients with low-grade *IDH1/2-*mutant gliomas and 52% of patients with high-grade *IDH1/2-*mutant gliomas have been assessed for *MGMT*-methylation status. Further, amongst *IDH1/*2-wild-type tumors, where *MGMT-*methylation status is instrumental for therapeutic selection, almost a fifth of high-grade gliomas and more than 3-quarters of low-grade gliomas were not assessed for *MGMT* status. This highlights the need for more widespread *MGMT* molecular profiling to identify patients who may benefit from available therapies.

Correlation analysis and genomic distance measurements further highlighted the strong association between molecular alterations, histopathologic grade, and patient survival across glioma subtypes. High-grade oligodendrogliomas (grade 3) and *IDH1/2-*mutant astrocytomas (grade 4) showed a higher mutational burden and distinct survival profile compared to low-grade oligodendrogliomas (grade 2) and astrocytomas (grades 2/3). In comparison, the relative molecular homogeneity and similar survival between grade 2 and grade 3 *IDH1/2-*mutant astrocytomas underscore the lack of well-defined features that can reliably distinguish across these grades. While there were some similarities in their mutational profiles, each glioma subtype demonstrated unique molecular characteristics. Glioblastoma featured several well-known alterations that largely segregated into distinct tumorigenesis pathways. For example, in glioblastoma, *CDKN2A/B* alterations were mutually exclusive from many alterations in the p53 and cell cycle pathways (eg, *TP53, MDM2/4, CDK4/6, and RB1*), both of which include *CDKN2A/B*. By contrast, *IDH1/2-*mutant astrocytomas had a broader range of cooccurring deleterious alterations across various pathways, including between *CDKN2A/B* and *EGFR*. Despite these associations, grade 4 *IDH1/2*-mutant astrocytomas demonstrated significant genomic distance from glioblastomas, reinforcing their distinct categorization from glioblastoma. The unique mutational profile for each glioma subtype supports the notion that a molecularly driven classification system for gliomas can enhance precision and improve the correlation between molecular characteristics and clinical behavior.

Although we characterized prognostic indicators for glioblastoma, *IDH1/2*-mutant astrocytoma, and oligodendroglioma, we were unable to comprehensively analyze molecular features for the pediatric-type and other *IDH1/2*-wild-type gliomas given the small sample sizes. To better understand the genomic drivers and survival differences for these less common glioma subtypes, a well-powered cohort study is necessary. These tumors demonstrate an aggressive clinical course reminiscent of *IDH1/2*-wild-type glioblastoma, with a median overall survival of 20.4 months in our cohort, similar to that of *IDH1/2*-wild-type glioblastoma.^3^ Orthogonal technologies, such as DNA methylation profiling may further distinguish the character of other *IDH1/2*-wild-type gliomas, with a study demonstrating overlapping methylation signatures of other *IDH1/2*-wild-type gliomas with *IDH1/2-*wild-type glioblastomas.^34^

Several limitations exist in this analysis, many of which are inherent in using large retrospective datasets. Errors in data entry and storage within large repositories may have influenced the clinical and molecular information used in our cohort, despite our efforts to manually update all available institutional data to extend follow-up length and verify molecular and treatment information. Additionally, as the molecular data was collected over 2 decades, there was heterogeneity in the coverage of certain genes and differences in how mutations were detected. This includes the assays and platforms used, some of which can impact the reporting of data such as heterozygous and homozygous copy number variation. Variations in the methodology used for *MGMT*-methylation profiling, both across cohorts and within cohorts, can contribute to the reported frequency of promoter methylation and introduce inaccuracy. Batch effects and germline filtering pipelines could also introduce variability in the reported frequency of molecular alterations. These factors limited the number of samples with complete molecular data that could be used for survival prognostication. While we aimed to capture as many clinical and molecular variables as possible, other factors may be present contributing to the survival difference across TCGA and non-TCGA cohorts. Finally, in parallel with limitations in molecular analysis, the availability of patient survival data limited the number of patients that could be included in multivariate modeling. Working toward robust data annotation in public datasets remains a goal which will strengthen our ability to leverage large, heterogeneous sources of data to define stable and clinically meaningful molecular markers.

Despite these considerations, we believe that these analyses provide updated survival benchmarks for the development of future clinical trials. The unified resource presented here helps decode the significance of molecular alterations in adult gliomas, serving as a guidepost for patients and healthcare providers alike.

## Supplementary material

Supplementary material is available online at *Neuro-Oncology* (https://academic.oup.com/neuro-oncology).

noae164_suppl_Supplementary_Materials

## Data Availability

Data used as part of this study from TCGA and GENIE are available online through public repositories. De-identified data collected from DFCI is publicly available as part of GENIE.
