# Supplementary material for: Contemporary prognostic signatures and refined risk stratification of gliomas: An analysis of 4400 tumors
Source: Neuro Oncol. 2024 Aug 21;27(1):195–208. doi: 10.1093/neuonc/noae164 (PMC11726335; doi:10.1093/neuonc/noae164)
Supplement: noae164_suppl_Supplementary_Materials [file noae164_suppl_supplementary_materials.docx]

Table of Contents

[Supplement 1 – Cohort Extraction and Sequencing 2](#_Toc168815541)

[Supplement 2 – Histogram of Year of Glioma Sample Collection 3](#_Toc168815542)

[Supplement 3 – Glioma Mutational Status 4](#_Toc168815543)

[Supplement 4 – Pathways Affected Across Glioma Subtypes 6](#_Toc168815544)

[Supplement 5 – Genomic Distances across Glioma Subtypes 7](#_Toc168815545)

[Supplement 6 – Demographic Breakdown Between Non-TCGA and TCGA Cohorts 8](#_Toc168815546)

[Supplement 7 – Glioma Survival by Grade: Non-TCGA Cohort 11](#_Toc168815547)

[Supplement 8 – CDKN2A/B and Chromosome 21q Loss in Glioblastoma 12](#_Toc168815548)

[Supplement 9 – Multivariate Adjusted Prognostic Features: DFCI Cohort 13](#_Toc168815549)

[Supplement 10 – Prognostic Signatures in IDH1/2-mutant Astrocytoma 14](#_Toc168815550)

[Supplement 11 – Internal Validation of Overall Survival Multivariate Models 15](#_Toc168815551)

# **Supplement 1 – Cohort Extraction and Sequencing**

**TCGA:**

**Clinical and molecular data for 1,020 gliomas collected between 1989-2013 were downloaded from the National Cancer Institute (NCI) Genomic Data Commons (TCGA-Low Grade Glioma and TCGA-Glioblastoma datasets, https://gdc.cancer.gov). All TCGA data provided mutational status (spanning 846 genes), copy number variants (CNV), and structural variants (SV) for included glioma samples, which was determined through whole exome sequencing. *MGMT* methylation was assessed using the Illumina GoldenGate assay, a microbead-based array.**

**Germline variants were filtered out from samples using tumor-matched sequencing data. Chromosomal arm level calls were generated through the Genomic Identification of Significant Targets in Cancer (GISTIC) module.**

**DFCI/BWH:**

**2,090 glioma samples from DFCI/BWH were assessed between 1993-2020. Clinical data and *MGMT* methylation status were extracted through retrospective review of the electronic medical record. Patient follow-up was collected through July 1, 2023. Through the DFCI-Profile initiative, targeted next-generation sequencing (OncoPanel) was performed at the Center for Advanced Molecular Diagnostics at Brigham and Women’s Hospital. OncoPanel results include mutational status, CNVs, and SVs. The number of genes assayed varied based on OncoPanel version, spanning from 277 (OncoPanel V1), 302 (OncoPanel V2), or 477 genes (OncoPanel V3). *MGMT* methylation was assessed using methylation-specific real-time quantitative polymerase chain reaction.**

**As OncoPanel sequencing was performed on glioma samples without matched normal DNA from patients, an internally developed pipeline was applied for germline variant filtering. Variants were first filtered out if their allele frequency was >0.1% in the Genome Aggregation Database (https://gnomad.broadinstitute.org) or if annotated as benign in the NIH ClinVar database (https://ncbi.nlm.nih.gov/clinvar). In the second step, these filtered variants were cross-referenced and added back if present in the Catalogue of Somatic Mutations in Cancer (COSMIC, https://cancer.sanger.ac.uk/cosmic). Chromosomal arm-level calls were generated using the Arm-level Copy-number Events in Targeted Sequencing (ASCETS, Spurr et al., 2020:** <https://doi.org/10.1093/bioinformatics/btaa980>**) platform developed at DFCI. Chromosome arms were considered amplified or deleted if more than 70% of the chromosome arm was altered.**

**GENIE (v10):**

Clinical and molecular data for 7,271 gliomas were downloaded from Synapse (17 institutions contributing glioma specific data, https://synapse.org/genie). Samples from DFCI/BWH were filtered out. A total of 32 distinct targeted-sequencing gene panels were applied across the glioma samples included in the filtered GENIE repository. 12 of these gene panels provided CNVs and 9 provided SVs. *IDH1/2*-wildtype and *H3*-wildtype gliomas were excluded if sufficient molecular data was not present to determine if it was an *IDH1/2*-wildtype glioblastoma. Applying this restriction resulted in glioma samples from 1986-2020, which were surveyed on 30 distinct gene panels, representing 45-760 genes. *MGMT* methylation was reported on a subset of GENIE samples, with samples undergoing either real-time quantitative polymerase chain reaction or pyrosequencing profiling. Reported “borderline” methylation was considered negative.

Germline variants were filtered out by an internal pipeline at the American Association of Cancer Research as well as by the institutions contributing data to GENIE. Chromosomal arm level calls were generated using ASCETS using the same sample amplification and deletion definition as DFCI/BWH. *MGMT* methylation data was reported and available only for a subset of GENIE gliomas, specifically those collected and reported by Memorial Sloan Kettering Cancer Center. Patient follow-up was updated using GENIE v13 released in April 2023 on Synapse.

# **Supplement 2 – Histogram of Year of Glioma Sample Collection**

**
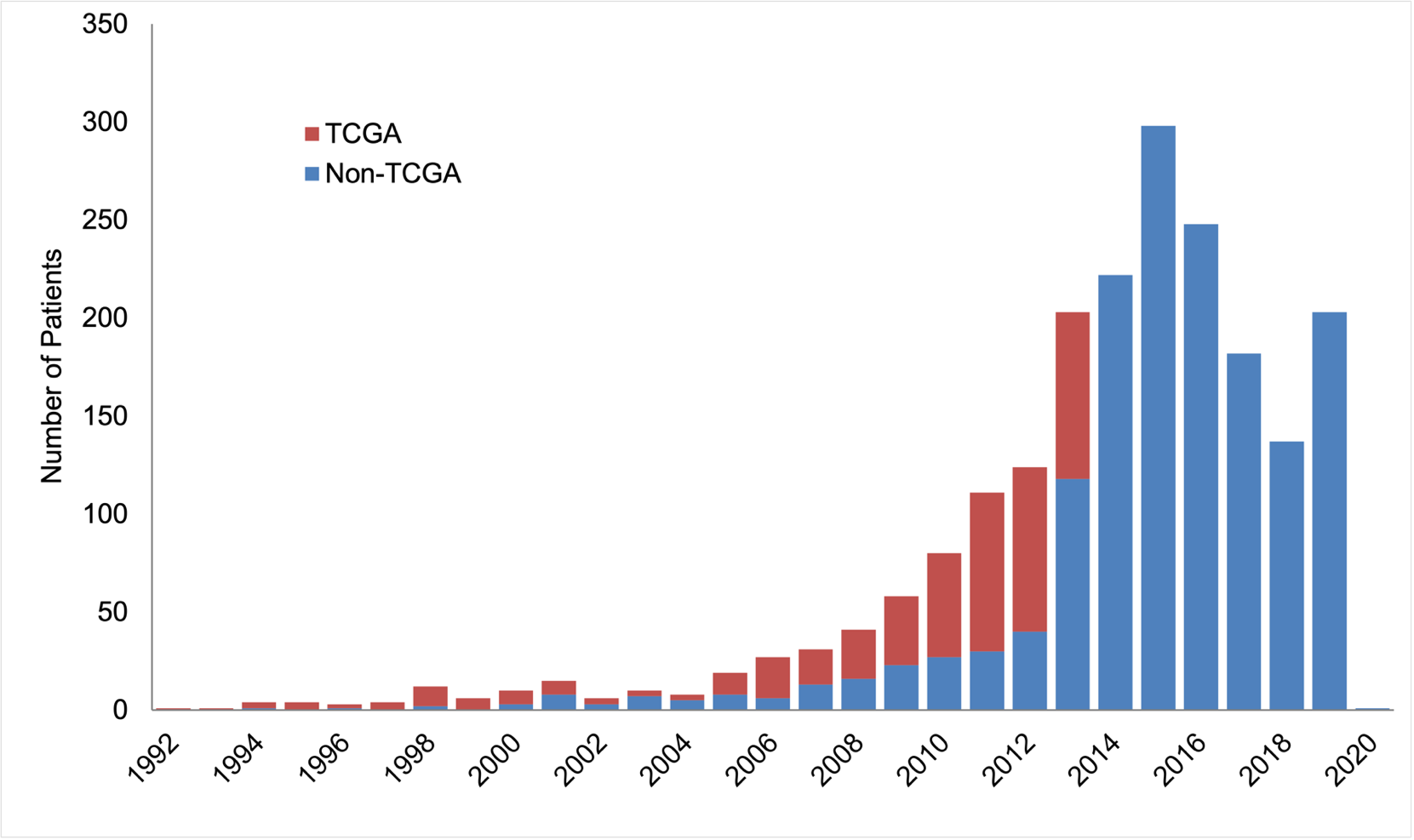
**

**Supplement 2:** Stacked bar plot of year of glioma sample collection for non-TCGA and TCGA patients included in analysis of survival and prognostic molecular features. Date of sample collection was inferred from the year of surgery; if this was not available, year of diagnosis was used.

# **Supplement 3 – Glioma Mutational Status**


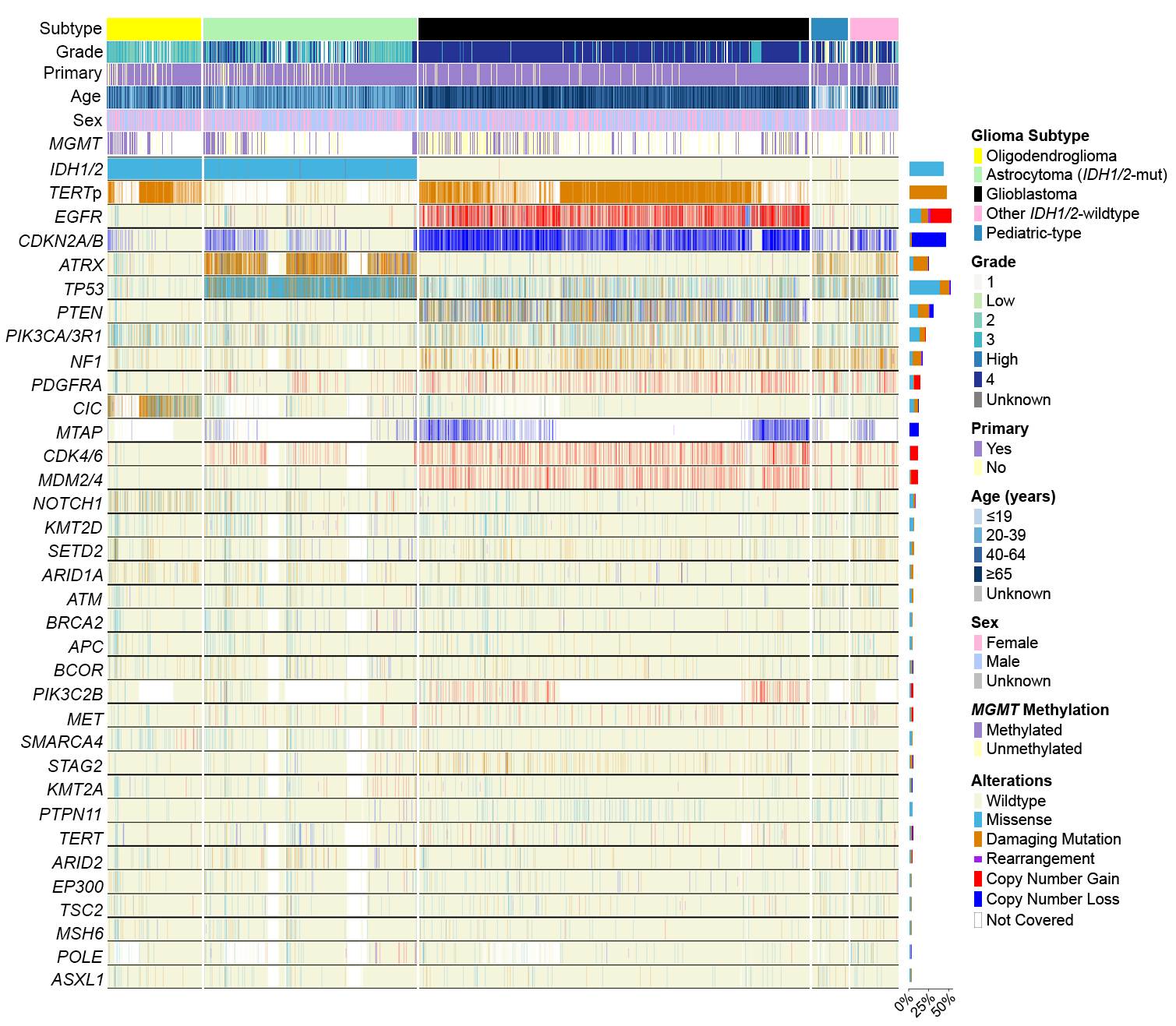


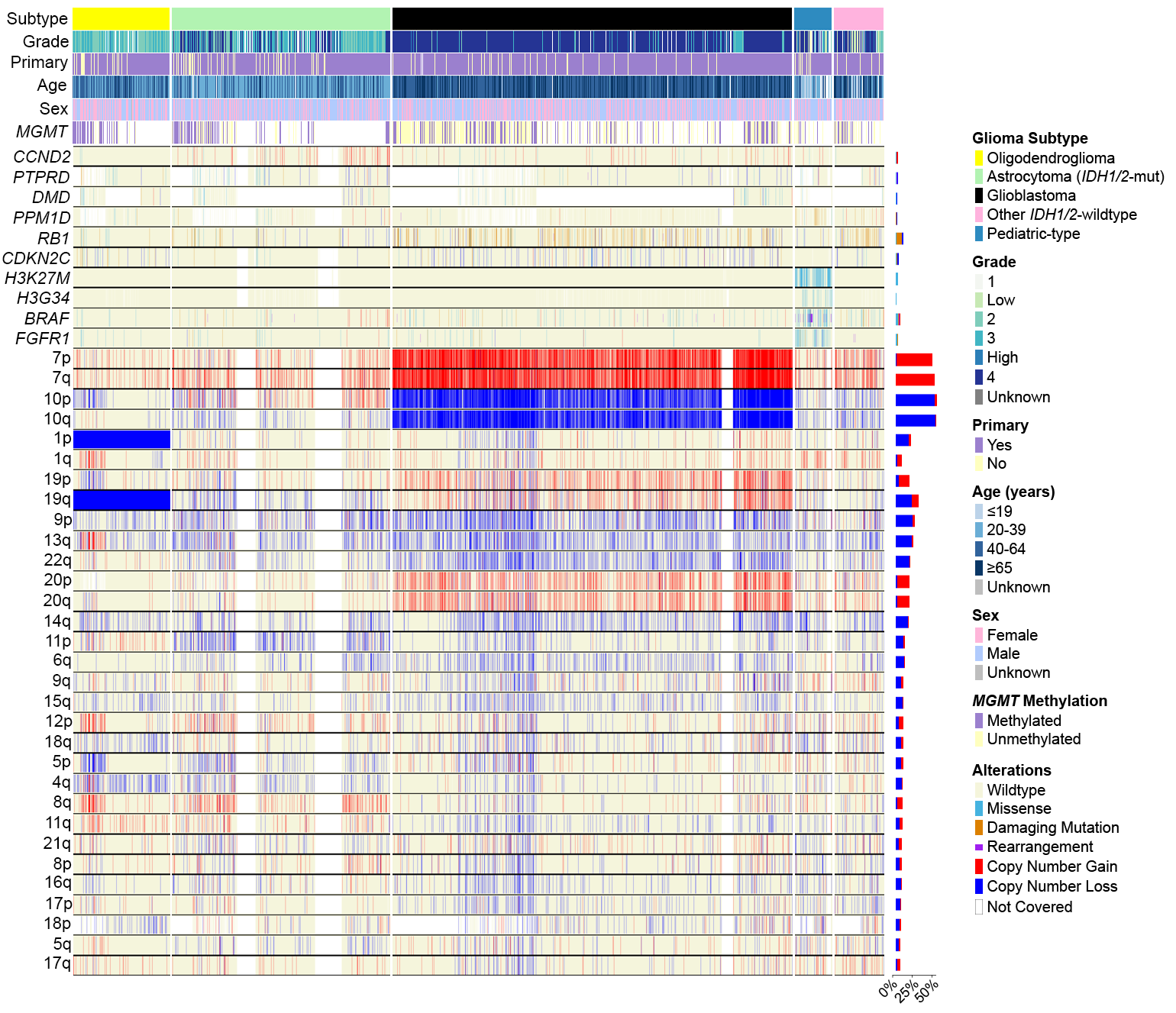


**Supplement 3:** Oncoprint showing alterations in genes which were altered in 5% of samples of any of the 5 major glioma subtypes or 4% of the entire study cohort. Additionally, arm-level chromosomal alterations shown if altered in ≥10% of the total cohort, ≥20% of a glioma subtype, or if the sum of the proportion of arms altered across glioma subtypes is ≥20%.

# **Supplement 4 – Pathways Affected Across Glioma Subtypes**

**
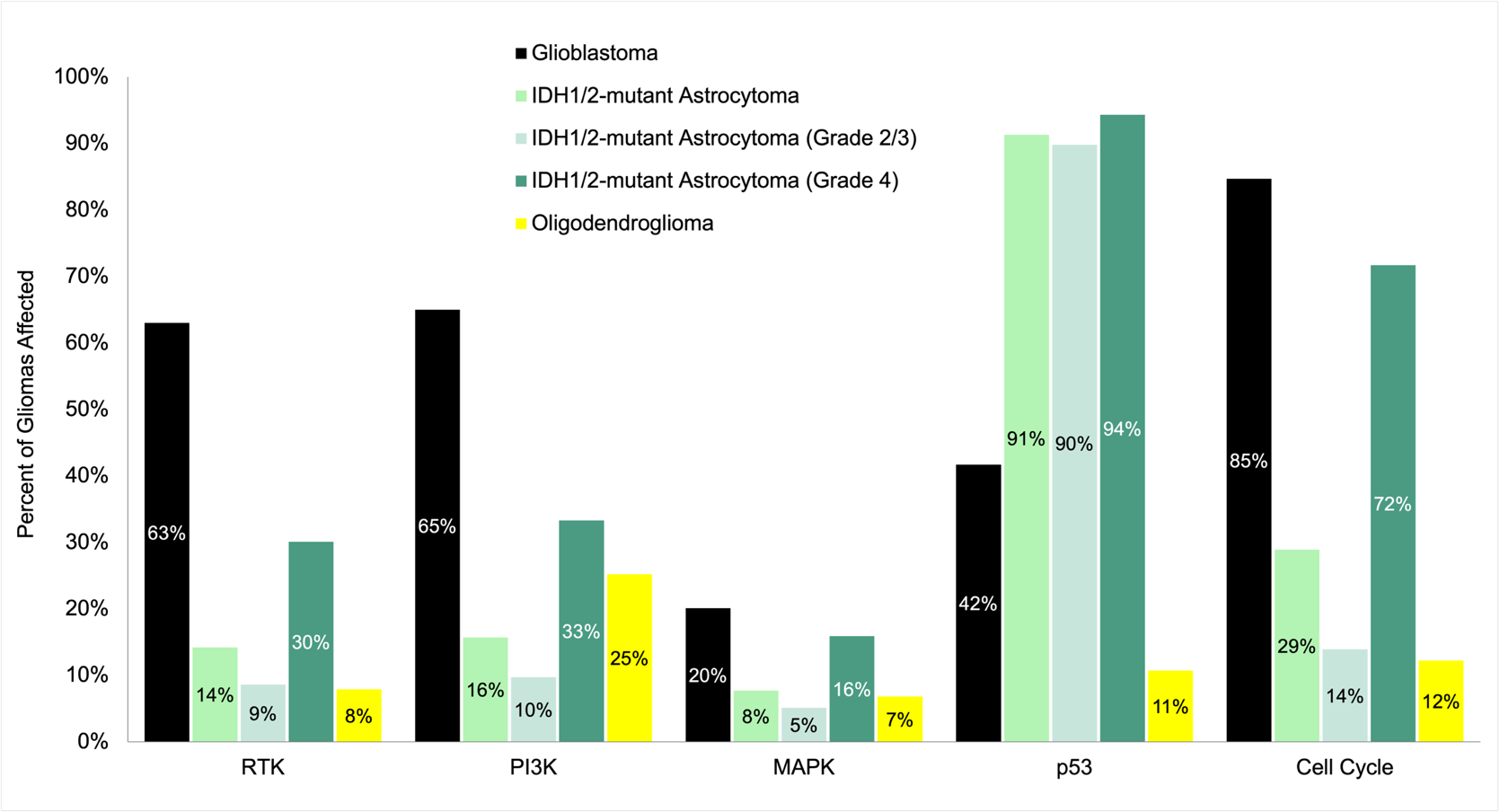
**

**Supplement 4:** Proportion of gliomas with an affected tumorigenic pathway as defined by select canonical genes in each pathway (as shown in Figure 2): receptor tyrosine kinase (RTK), phosphoinositide-3-kinase (PI3K), mitogen-activated protein kinase (MAPK), p53, and cell cycle.

# **Supplement 5 – Genomic Distances across Glioma Subtypes**

**Supplement 5:** Jaccard distances quantifying genomic difference between (A) glioblastoma and *IDH1/2*-mutant astrocytoma, (B) glioblastoma and oligodendroglioma, and (C) *IDH1/2*-mutant astrocytoma and oligodendroglioma. p<0.001 (***)

# **Supplement 6 – Demographic Breakdown Between Non-TCGA and TCGA Cohorts**

| Variable | Non-TCGA | | TCGA | p-value |
| --- | --- | --- | --- | --- |
| Patients, n (%) | 1870 | | 325 |  |
| DFCI/BWH | 791 (42.3) | | 0 (0.0) |  |
| GENIE (v10) | 1079 (57.7) | | 0 (0.0) |  |
| Sex (Female), n (%) | 763 (40.8) | | 123 (37.8) | p=0.34 |
| Median Age, years (range) | 61.0 (5.5-94.0) | | 61.1 (23.5-89.3) | p=0.55 |
| Primary Tumor, n (%) | 1733 (92.8) | | 325 (100.0) | p<0.001 |
| Age (years), n (%) |  | |  | p=0.82 |
| ≤19 | 5 (0.3) | | 0 (0.0) |  |
| >19-39 | 48 (2.6) | | 8 (2.5) |  |
| 40-64 | 1095 (59.0) | | 191 (58.8) |  |
| ≥65 | 709 (38.2) | | 126 (38.8) |  |
| Race (White), n (%) | 1624 (93.2) | | 292 (90.7) | p=0.15 |
| Histopathologic Diagnosis, n (%) | |  |  | p<0.001 |
| Glioblastoma | 1661 (88.8) | | 257 (79.1) |  |
| Astrocytoma | 132 (7.0) | | 44 (13.5) |  |
| Oligodendroglioma | 2 (0.1) | | 11 (3.4) |  |
| Other Gliomas | 75 (4.0) | | 13 (4.0) |  |
| Molecular Alterations, n (%) |  | |  |  |
| *TERT* promoter | 1444 (90.9) | | 68 (94.4) | p=0.42 |
| *EGFR* amplification | 841 (45.3) | | 197 (60.6) | p<0.001 |
| Whole Chr7 Gain/Chr10 loss | 956 (53.2) | | 272 (83.7) | p<0.001 |
| *CDKN2A/B* hom. del. | 1065 (57.6) | | 165 (50.8) | p=0.03 |
| *PDGFRA* | 198 (10.7) | | 48 (14.8) | p=0.04 |
| *PTEN* | 910 (49.0) | | 135 (41.5) | p=0.02 |
| *ATRX* | 48 (2.6) | | 9 (2.8) | p=1.00 |
| *TP53* | 469 (25.3) | | 69 (21.2) | p=0.14 |

**Demographics of Glioblastoma Patients Across Cohorts**

**Demographics of *IDH1/2*-mutant Astrocytoma Patients Across Cohorts**

| Variable | Non-TCGA | | TCGA | p-value |
| --- | --- | --- | --- | --- |
| Patients, n (%) | 932 | | 266 |  |
| DFCI/BWH | 358 (38.4) | | 0 (0.0) |  |
| GENIE (v10) | 574 (61.6) | | 0 (0.0) |  |
| Grade, n (%) |  | |  | p<0.001 |
| Grade 2 | 140 (15.0) | | 126 (47.4) |  |
| Grade 3 | 311 (33.4) | | 116 (43.6) |  |
| Grade 4 | 311 (33.4) | | 23 (8.6) |  |
| High | 9 (1.0) | | 0 (0.0) |  |
| Low | 6 (0.6) | | 0 (0.0) |  |
| Unknown | 155 (16.6) | | 1 (0.4) |  |
| Sex (Female), n (%) | 364 (39.1) | | 118 (44.4) | p=0.14 |
| Median Age, years (range) | 37.0 (10.9-90.0) | | 37.0 (14.4- 74.0) | p=0.87 |
| Primary Tumor, n (%) | 756 (81.3) | | 226 (100.0) | p<0.001 |
| Age (years), n (%) |  | |  | p=0.16 |
| ≤19 | 24 (2.6) | | 1 (0.4) |  |
| <19-39 | 503 (54.5) | | 152 (57.1) |  |
| 40-64 | 369 (40.0) | | 105 (39.5) |  |
| ≥65 | 27 (2.9) | | 8 (3.0) |  |
| Race (White), n (%) | 763 (89.8) | | 249 (94.7) | p=0.02 |
| Histopathologic Diagnosis, n (%) | |  |  | p<0.001 |
| Glioblastoma | 274 (29.4) | | 23 (8.6) |  |
| Astrocytoma | 532 (57.1) | | 127 (47.7) |  |
| Oligodendroglioma | 41 (4.4) | | 40 (15.0) |  |
| Other Gliomas | 85 (9.1) | | 76 (28.6) |  |
| Molecular Alterations, n (%) |  | |  |  |
| *TERT* promoter | 32 (6.1) | | 8 (5.4) | p=0.91 |
| *EGFR* amplification | 14 (2.0) | | 3 (1.1) | p=0.54 |
| Whole Chr7 Gain/Chr10 loss | 4 (0.6) | | 4 (1.5) | p=0.31 |
| *CDKN2A/B* homozygous deletion | 90 (12.6) | | 9 (3.4) | p<0.001 |
| *PDGFRA* | 60 (6.4) | | 17 (6.4) | p=1.00 |
| *PTEN* | 36 (3.9) | | 4 (1.5) | p=0.09 |
| *ATRX* | 525 (56.9) | | 174 (65.4) | p=0.02 |
| *TP53* | 865 (93.0) | | 232 (87.2) | p<0.01 |

**Demographics of *IDH1/*2-mutant Oligodendroglioma Patients Across Cohorts**

| Variable | Non-TCGA | | TCGA | p-value |
| --- | --- | --- | --- | --- |
| Patients, n (%) | 367 | | 164 |  |
| DFCI/BWH | 176 (48.0) | | 0 (0.0) |  |
| GENIE (v10) | 191 (52.0) | | 0 (0.0) |  |
| Grade, n (%) |  | |  | p<0.001 |
| Grade 2 | 149 (40.6) | | 91 (55.5) |  |
| Grade 3 | 157 (42.8) | | 73 (44.5) |  |
| High | 2 (0.5) | | 0 (0.0) |  |
| Low | 3 (0.8) | | 0 (0.0) |  |
| Unknown | 56 (15.3) | | 0 (0.0) |  |
| Sex (Female), n (%) | 177 (48.4) | | 73 (44.5) | p=0.47 |
| Median Age, years (range) | 43.3 (12.5-81.0) | | 45.1 (17.8- 75.8) | p=0.27 |
| Primary Tumor, n (%) | 266 (72.5) | | 164 (100.0) | p<0.001 |
| Age (years), n (%) |  | |  | p=0.68 |
| ≤19 | 5 (1.4) | | 1 (0.6) |  |
| >19-39 | 129 (35.3) | | 59 (36.2) |  |
| 40-64 | 204 (55.9) | | 87 (53.4) |  |
| ≥65 | 27 (7.4) | | 16 (9.8) |  |
| Race (White), n (%) | 312 (90.4) | | 151 (95.0) | p=0.29 |
| Histopathologic Diagnosis, n (%) | |  |  | p<0.001 |
| Glioblastoma | 2 (0.5) | | 0 (0.0) |  |
| Astrocytoma | 8 (2.2) | | 6 (3.7) |  |
| Oligodendroglioma | 328 (89.4) | | 122 (74.4) |  |
| Other Gliomas | 29 (7.9) | | 36 (22.0) |  |
| Molecular Alterations, n (%) |  | |  |  |
| *TERT* promoter | 234 (93.6) | | 83 (96.5) | p=0.46 |
| *EGFR* amplification | 0 (0.0) | | 0 (0.0) | p=NA |
| Whole Chr7 Gain/Chr10 loss | 1 (0.3) | | 0 (0.0) | p=1.00 |
| *CDKN2A/B* homozygous deletion | 8 (2.2) | | 0 (0.0) | p=0.13 |
| *PDGFRA* | 17 (4.6) | | 3 (1.8) | p=0.19 |
| *PTEN* | 10 (2.7) | | 2 (1.2) | p=0.45 |
| *ATRX* | 30 (8.2) | | 3 (1.8) | p=0.01 |
| *TP53* | 33 (9.0) | | 6 (3.7) | p=0.046 |

**Supplement 6:** Demographic breakdown of non-TCGA and TCGA cohorts for patients with glioblastoma, *IDH1/2*-mutant astrocytoma, and *IDH1/2*-mutant oligodendroglioma.

# **Supplement 7 – Glioma Survival by Grade: Non-TCGA Cohort**

**Supplement 7:** Kaplan-Meier curves for overall survival, stratified by grade, for patients in the non-TCGA cohort with (A) oligodendroglioma, (B) *IDH1/2-*mutant astrocytoma, and (C) other *IDH1/2*-wildtype gliomas. Peds-type: LG: low-grade pediatric-type gliomas, DA/NEC: *IDH1/*2-wildtype diffuse astrocytic gliomas/”Not Elsewhere Classified”, Peds-type: HG: high-grade pediatric-type gliomas.

# **Supplement 8 – *CDKN2A/B* and Chromosome 21q Loss in Glioblastoma**

**Supplement 8:** (A) Kaplan-Meier curves for overall survival in patients with glioblastoma, stratified by *CDKN2A/B* status, demonstrate similar reduction in survival between patients with heterozygous or homozygous *CDKN2A/B* loss versus patients with intact *CDKN2A/B*. (B) Kaplan-Meier curves for overall survival in patients with glioblastoma, stratified by loss or retention of chromosome 21q, show 21q loss positively influences survival. *CDKN2A/B* +/-: heterozygous loss, *CDKN2A/B* -/-: homozygous loss.

# **Supplement 9 – Multivariate Adjusted Prognostic Features: DFCI Cohort**


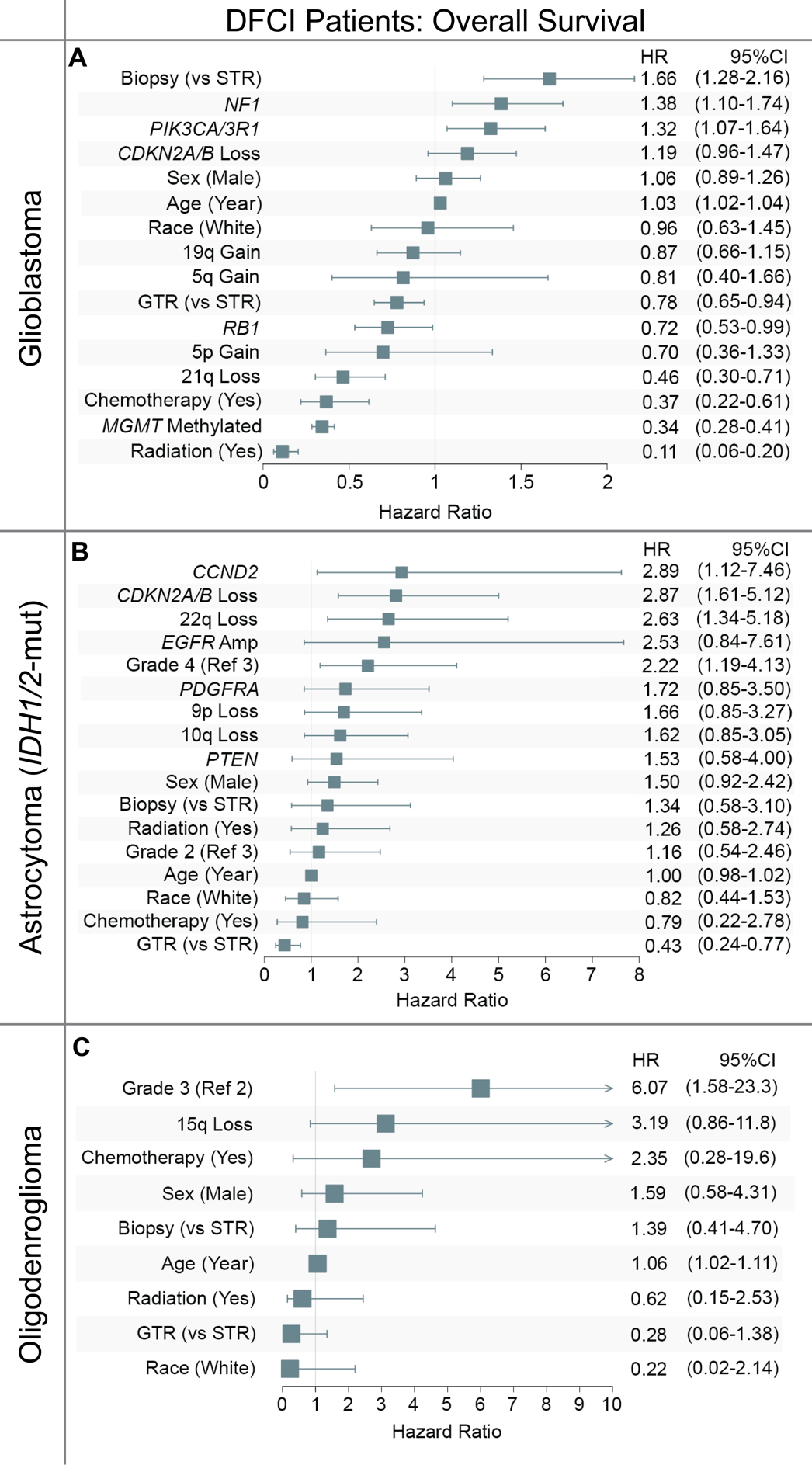


**Supplement 9:** Multivariate adjusted hazard ratios and 95% confidence intervals (CI) show differential features for overall survival across the DFCI cohort for (A) glioblastoma, (B) *IDH1/2*-mutant astrocytoma, and (C) oligodendroglioma. Model adjusts for extent of resection and receipt of radiation. GTR: gross-total resection, STR: sub-total resection.

# **Supplement 10 – Prognostic Signatures in *IDH1/2*-mutant Astrocytoma**

**Supplement 10:** Patients with *IDH1/2*-mutant astrocytomas stratified by (A) *EGFR* amplification, (B) *CDKN2A/B* homozygous and heterozygous loss, (C) 10q loss, and (D) 22q loss show significantly worse overall survival with each of these prognostic features on Kaplan-Meier curves. (E) Alteration status of *IDH1/2-*mutant astrocytomas with either *EGFR* amplification, *CDKN2A/B* loss, 10q loss, and/or 22q loss, show limited co-occurrence of these four negative prognostic features. *CDKN2A/B* +/-: heterozygous loss, *CDKN2A/B* -/-: homozygous loss.

# **Supplement 11 – Internal Validation of Overall Survival Multivariate Models**

***IDH1/2*-Mutant Astrocytoma**

**Glioblastoma**


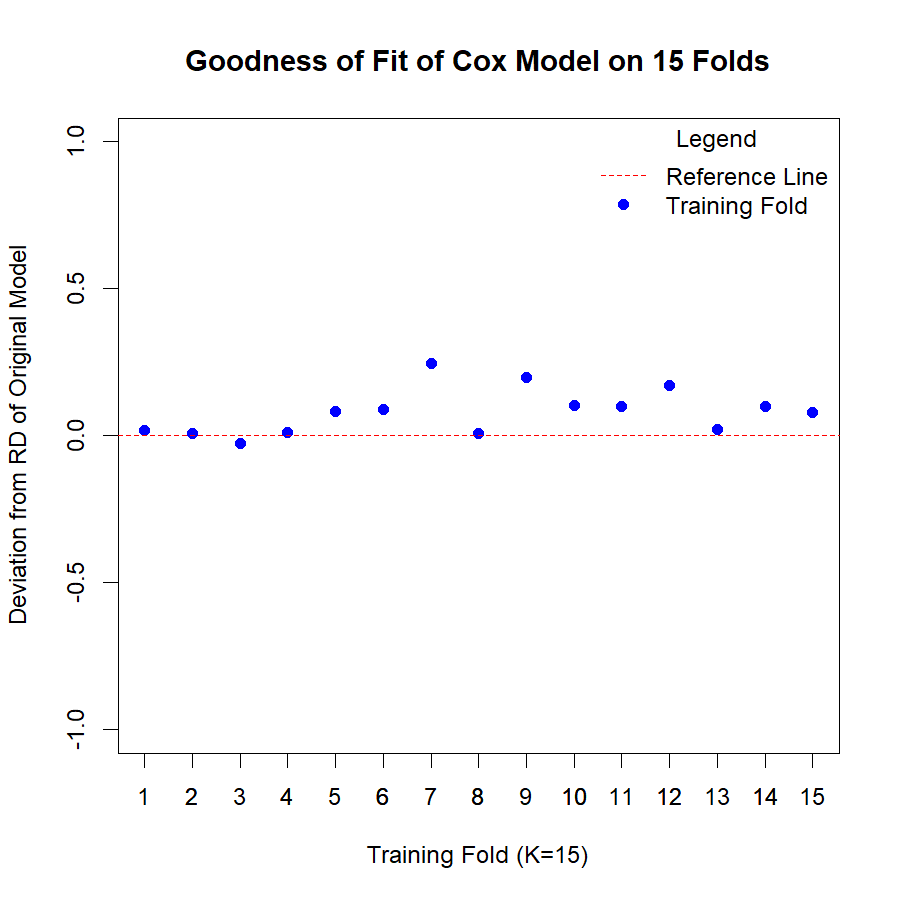

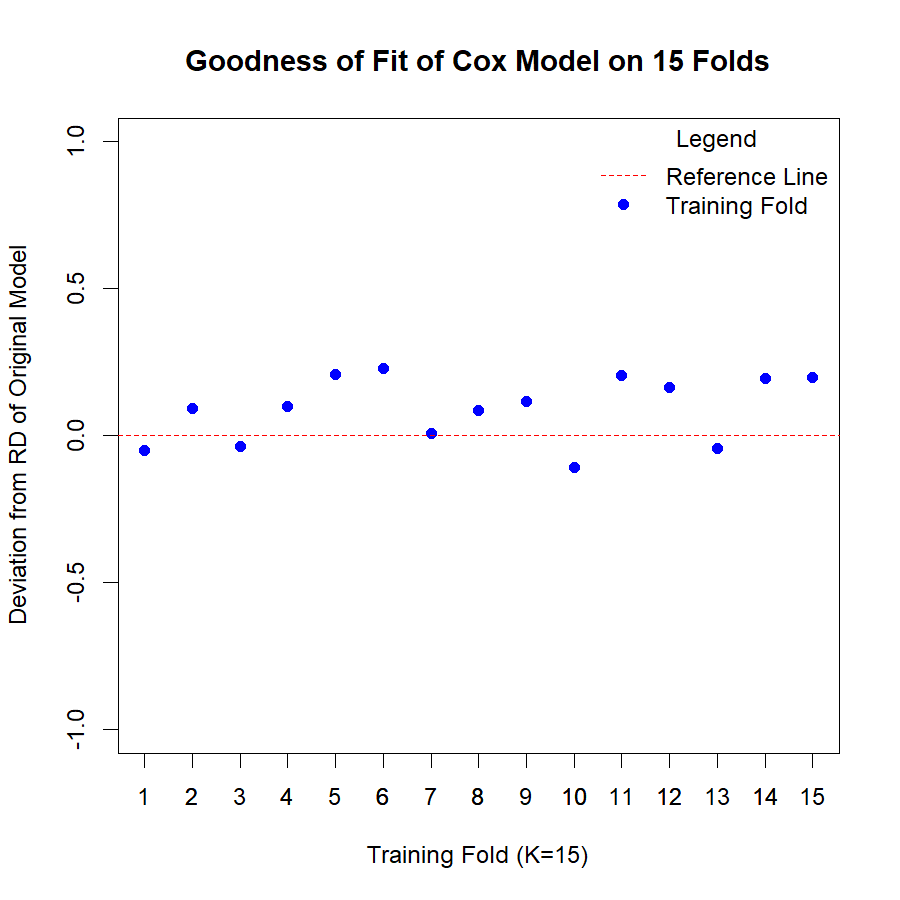


***IDH1/2*-Mutant Oligodendroglioma**


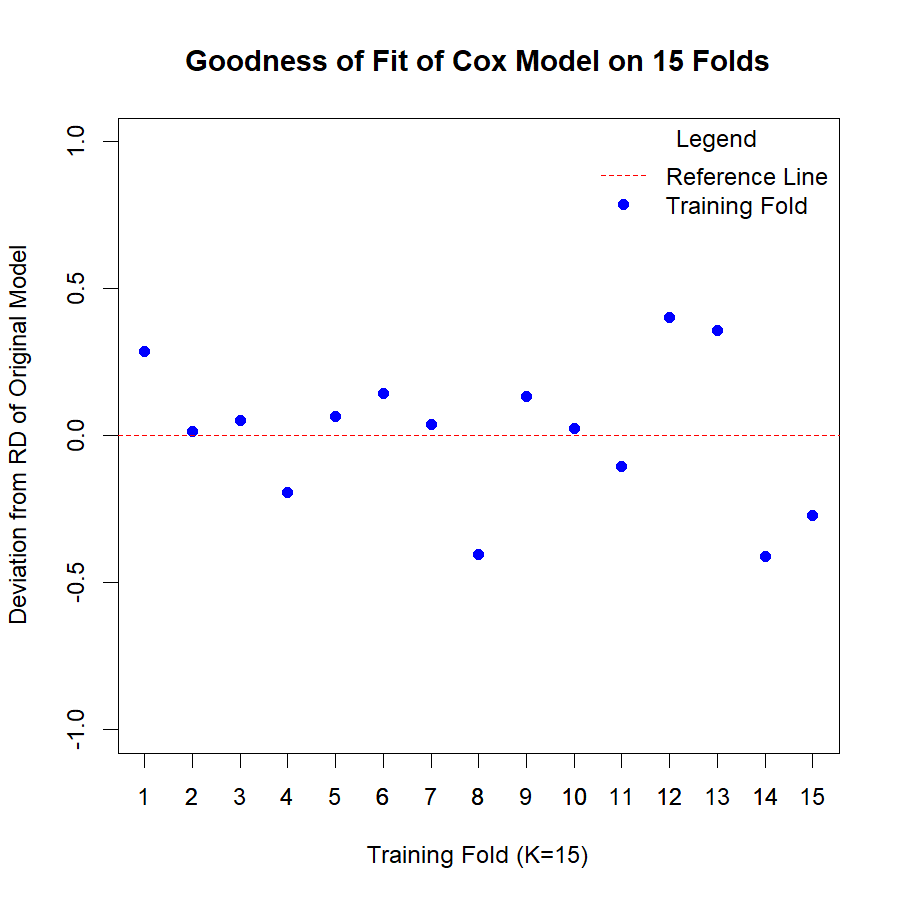


**Supplement 11:** Pseudo-R^2^ values (RD) across each fold (randomly selected sample, 65% of whole dataset) compared to the RD value of the multivariate model performed on the entire cohort. Folds were constructed separately for glioblastoma, *IDH1/2*-mutant astrocytoma, and *IDH1/2*-mutant oligodendroglioma to enable comparison of multivariate models and molecular/clinical features. Median RD difference for glioblastoma: 0.08, for *IDH1/2*-mutant astrocytoma: 0.11, for *IDH1/2*-mutant oligodendroglioma: 0.14.
